# Assessment of healthy behaviors for metabolic syndrome among Korean adults: a modified information-motivation-behavioral skills with psychological distress

**DOI:** 10.1186/s12889-016-3185-8

**Published:** 2016-06-18

**Authors:** Guna Lee, Sook Ja Yang, Yeon Kyung Chee

**Affiliations:** College of Nursing, Ewha Womans University, Seoul, South Korea; Department of Child Development, Ewha Womans University, Seoul, South Korea

**Keywords:** Metabolic syndrome, Healthy behavior, Information-motivation-behavioral skills model (IMB), Psychological Distress

## Abstract

**Background:**

Since the worldwide incidence of metabolic syndrome (Mets) has rapidly increased, healthy behaviors such as weight control, engaging in physical activity, and healthy diet have been crucial in the management of Mets. The purpose of this study was to examine healthy behaviors practice and factors that affect the practice in relation to Mets on the basis of a modified Information-Motivation-Behavioral skills model (IMB) with psychological distress, which is a well-known factor affecting healthy behaviors among individuals with Mets.

**Methods:**

Study participants were 267 community dwelling adults (*M* age: 54.0 ± 8.1 years) with Mets who were attending public health centers located in Seoul, South Korea. A structured questionnaire was administered in the areas of information, motivation, behavioral skills, and practice of Mets healthy behaviors and levels of psychological distress from May 2014 to September 2014. Structural equation modeling was used to test the modified IMB model.

**Results:**

The modified IMB model had a good fit with the data, indicating that motivation and behavioral skills directly influenced the practice of Mets healthy behaviors, whereas information and psychological distress directly influenced motivation and influenced the practice of healthy behaviors through behavioral skills. These components of the modified IMB model explained 29.8 % of the variance in healthy behaviors for Mets.

**Conclusion:**

Findings suggested that strengthening motivation and behavioral skills for healthy behaviors can directly enhance healthy behavior practice. Providing information about Mets related healthy behaviors and strategies for psychological distress management can be used as the first line evidence based intervention to systemically enhance motivation and behavioral skills among individuals with Mets.

## Background

The global incidence of metabolic syndrome (Mets) is increasing and has impacted approximately 20–30 % of the adult population in Korea [1, 2]. Mets is defined by its clinical features including abdominal obesity, elevated triglycerides, blood pressure, plasma glucose, and reduced high density lipoprotein (HDL) [3–5]. Mets is known to be a risk factor associated with increased incidence of diabetes, hypertension, cerebrovascular and cardiovascular diseases, and various cancers, as well as overall mortality [6]. In individuals with Mets, risk for cardiovascular disease is reported to be twice as high as for healthy individuals [7], and risk for diabetes is reported to be 3.5–5 times higher [5]. Given these associations of Mets with serious complications, effective interventions are needed.

Unhealthy behaviors such as lack of physical activity and unbalanced diets combined with genetic factors, are primary causes of Mets [3–5]. Healthy behavioral interventions have been reported to have strong positive effects on abdominal obesity, elevated blood pressure, triglycerides, and blood glucose levels [8]. The worldwide guidelines for Mets [3–5] recommend that Mets treatments should include lifestyle improvement. Among healthy behaviors, increased muscle mass through regular physical activity improves Mets symptoms by reducing insulin resistance and weight control. Also, balanced dietary habits prevent Mets complications [5, 9]. For patients who are not taking Mets medications, in particular, healthy behaviors can be the most important intervention strategy [4, 5].

Modification of a life style requires consideration of influencing factors, including knowledge, attitude, social support, and self-efficacy, each associated with healthy behaviors [10, 11]. Previous studies showed that cognitive factors such as knowledge [12], emotional factors such as attitude toward healthy behaviors [13] or perceived social support [14], and behavioral skills factors such as self-efficacy [15, 16] positively influenced healthy behaviors in patients with Mets. Although the factors influencing healthy behavior for Mets are being widely studied [17], a lack persists in comprehensive evidence based studies that examine the importance and mechanism by which factors influence healthy behaviors for Mets. Empirical data are needed that assess healthy behaviors for Mets based on a theoretical framework that evaluates its relative cognitive, emotional, and behavioral skills aspects.

Although theories of healthy behaviors abound, including the theory of planned behavior [18] and the health promotion model [19], the information-motivation-behavioral skills (IMB) model is useful in that it explains factors influencing healthy behavior for Mets; cognitive, emotional, and behavioral skills factors [20, 21]. The IMBmodel conceptualizes factors influencing engagement in and continuation of healthy behaviors including information, motivation, and behavioral skills. Information and motivation influence behavioral skills and healthy behaviors and, in turn, behavioral skills influence healthy behaviors [20]. In the IMB model, information related to management of Mets and prevention of complications impacts not only motivation toward healthy behavior [21], but also enhancement of behavioral skills needed for healthy behaviors [22, 23] and increasing such healthy behaviors [12]. Motivation and behavioral skills are known to be predisposing factors for healthy behaviors for Mets [21]. Motivation increases healthy behaviors related to physical activities and dietary improvements through personal motivation [24] or positive social motivation provided by family, peers, and close relatives [25]. Moreover, behavioral skills play an important role in maintaining healthy behavior by voluntarily setting goals over a long period of time [14].

Psychological distress causes increased excretion of catecholamine and cortisol, which induce excessive appetite and elevated blood pressure that can lead to Mets [26, 27], and adds to the onset of Mets related complications such as diabetes and hypertension [28]. Researchers reported that higher stress levels resulted in decreased healthy behaviors, such as less exercise, poorer diet, and smoking [29]. Also, stress has a negative influence on motivation [30] and behavioral skills [31] and influences healthy behaviors through motivation [32] and behavioral skills [33], suggesting that management of stress is an essential part of healthy behaviors for Mets.

**Fig. 1:**
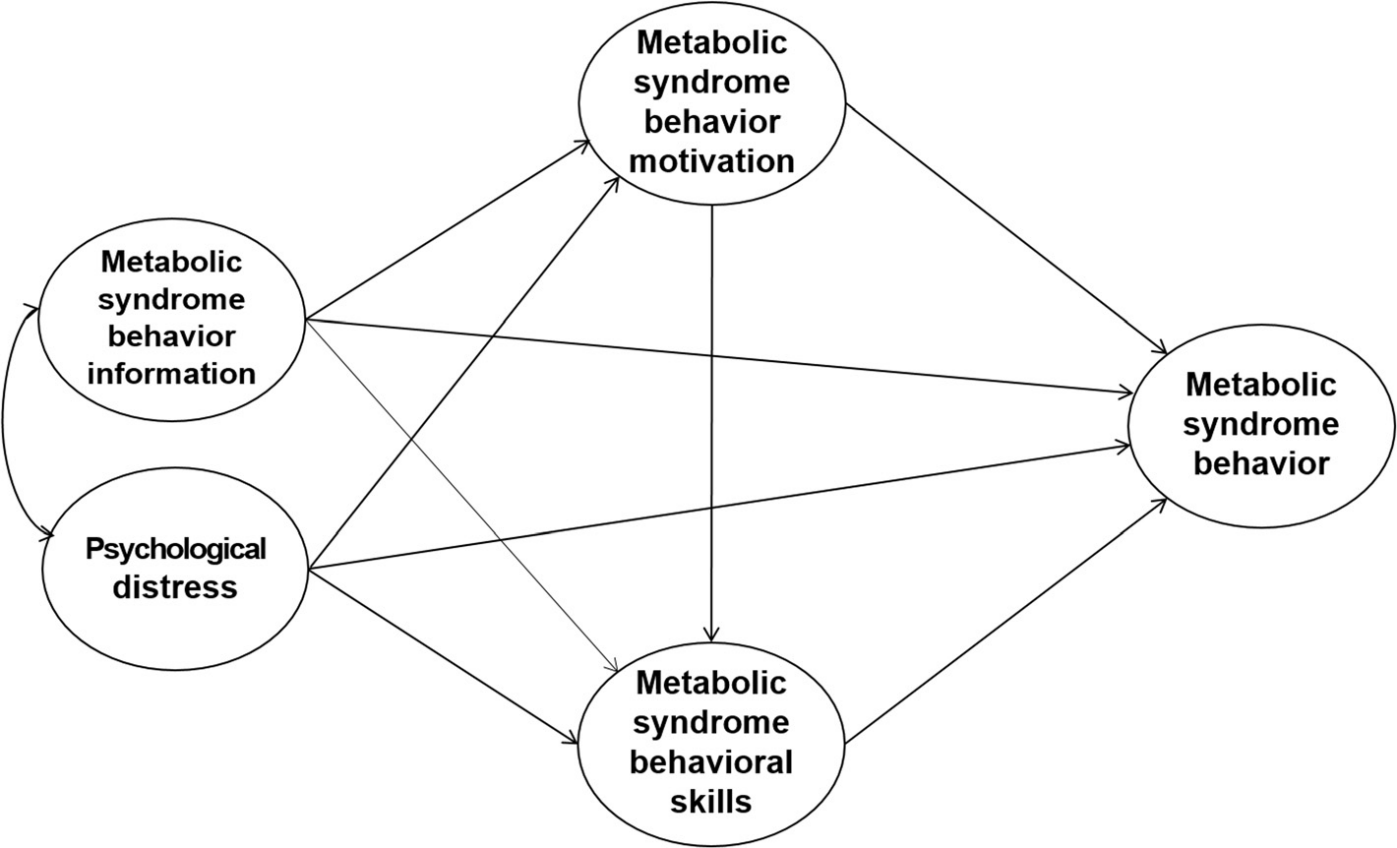
Modified Information-Motivation-Behavioral skill model of healthy behavior for metabolic syndrome

The purpose of this study was to test a causal model to explain and predict healthy behaviors for Mets using the IMB model, with the addition of psychological distress, which may affect healthy behavior in individuals with Mets. As an exogenous variable, the relationship between information and motivation toward healthy behaviors was modified as a causal relationship (Fig. 1). Understanding of the relationships among the cognitive, emotional, and behavioral skills factors that influence healthy behaviors for Mets in the IMB model can help in the development and implementation of evidence based interventions for Mets in the community.

## Methods

### Study participants

Study participants were adults aged 30 to 65, who had been diagnosed with Mets, based on the International Classification of Diseases [5] and had visited public health centers in Seoul. Because individuals under 30 years of age show low rates of prevalence of Mets [1] and those aged over 65 are less prone to behavioral changes [5], the criterion was designed to obtain information that reflects general characteristics of Mets and behavioral intervention. Also, adults who were not receiving pharmacological treatment and using only healthy behavioral treatment were eligible for enrollment in the study to identify the effects of healthy behaviors on Mets more accurately. The detailed eligibility criteria included those who (1) have three or more risk factors for Mets (abdominal obesity: waist size ≥ 90 cm for men and ≥ 80 cm for women; elevated blood pressure (BP) ≥ 130/85 mmHg; decreased HDL cholesterol < 40 mg/dl for men and < 50 mg/dl for women; elevated triglycerides ≥ 150 mg/dl; and elevated blood sugar with fasting blood glucose (FBG) ≥ 100 mg/dl); (2) are not currently taking medications for hypertension or diabetes; (3) understood the objectives of this study and provided consent to participate; (4) can communicate, and are capable of understanding and responding to the questionnaire; and (5) are ambulant and can practice healthy behaviors on their own.

Data were collected using convenience sampling at five public health centers in four districts in Seoul from May 2014 to September 2014. The study participants who agreed to participate in this study were selected and provided informed consent. For cases in which participants did not or could not write, the survey was conducted in a personal interview format by trained interviewers. Initially 300 questionnaires were administered, from which 267 were used for data analyses, after excluding data from 33 participants for unreliable answers (30 for missing answers and three for providing the same responses to every question).

### Measures

This study used a structured questionnaire to measure the sociodemographic characteristics of study participants (gender, age, education, employment status, and duration of Mets since diagnosis), psychological distress, and factors in the modified IMB model including information, motivation, behavioral skills, and the actual practice of healthy behaviors for Mets. Survey instruments that measured information, motivation, and behavioral skills of healthy behaviors for Mets were modified, in part, by the researchers, through testing of content validity. Content validity was examined by an expert panel that comprised professors of medicine, nursing, and human development. Then, reliability of the modified measures was evaluated based on a preliminary survey of 120 patients with Mets, and estimates of all measures by Cronbach’s alpha exceeded .70.

#### Information

Information consisted of 14 questions that assess knowledge and recognition of healthy behaviors for Mets [34]. Questions were divided into four domains, consisting of three questions on abdominal obesity information (e.g., Diet and exercise are effective for controlling obesity); four on elevated BP information (e.g., Hypertension can only be treated with medication); four on elevated triglycerides and low HDL cholesterol (e.g., Having lower levels of all types of cholesterol is good for health); and three on elevated blood glucose information with FBG (e.g., Regular exercise reduces blood glucose). Each correct answer was given 1 point, and no points were given to wrong answers or responses of “I don’t know,” with higher scores indicating having more information about healthy behaviors (Cronbach’s α = .72).

#### Psychological distress

Psychological distress was measured using the 5-item Brief Encounter Psychological Instrument, which has been used broadly in health examination [35, 36] on a 5-point Likert type scale (1 = never to 5 = always) that evaluated the extent to which participants felt stress in daily living for the past 1 month (e.g., In the past month, have you ever felt as if there are more demands on your life, emotionally and physically, than you can handle comfortably?) [35, 36]. The questions assessed extrinsic demand, intrinsic demand, attributional demand, demand uncertainty, and demand perspective. High scores indicated higher levels of psychological distress (Cronbach’s α = .83).

#### Motivation

Motivation consisted of 18 personal motivation questions about personal attitude toward healthy behavior for Mets and 18 social motivation questions about social support perceived by study participants in relation to Mets healthy behaviors. Each answer was scored on a 7-point Likert type scale with higher scores indicating higher motivation toward healthy behaviors [37–39]. Questions were categorized into nine aspects of healthy behaviors for Mets, including exercise, weight control, dietary improvement, moderate drinking, smoking cessation, adequate sleep, sufficient rest, stress management, and regular health examinations. Personal motivation (e.g., “I think weight control for management of Mets is~”) included questions on instrumental attitudes (1 = extremely worthless to 7 = extremely worthy) and empirical attitudes (1 = extremely unpleasant to 7 = extremely pleasant). Social motivation included normative beliefs (e.g., “People around me think weight control is necessary for management of Mets.”) and compliance motivations (e.g., “I agree with people around me thinking weight control is necessary for management of Mets.”) with responses ranging from 1 (strongly disagree) to 7 (strongly agree). (Cronbach’s α = .82 and .86 for personal and social motivation, respectively).

#### Behavioral skills

Behavioral skills for Mets consisted of 24 questions that evaluated behavioral skills (e.g., “I can exercise regularly.”) for weight control, dietary improvement, moderate drinking, smoking cessation, adequate sleep, enough rest, stress management, and regular health examinations using a 4-point Likert type format (1 = never to 4 = always) with higher scores indicating greater behavioral skills for healthy behaviors (Cronbach’s α = .92) [40, 41].

#### Healthy behavior

Healthy behavior was measured using a healthy behavior evaluation tool for Mets, comprising 35 questions on a 4-point Likert type scale (1 = never to 4 = always) with higher scores indicating healthy behaviors practiced to a greater degree [39]. The questions (e.g., I regularly exercise for 30 min a day, 5 or more times a week) consisted of 8 items on physical activities and weight control; 16 items on dietary habits; 3 items on drinking and smoking; 2 items on sleep and rest; 3 items on stress management; and 3 items on health examination. The questions were scored 4 points for “always,” 3 for “often,” 2 for “sometimes,” and 1 for “never” (Cronbach’s α = .93).

### Data analysis

Descriptive statistics and correlation analysis were performed to identify the characteristics of study participants and the factors of the modified IMB model using SPSS version 22.0. Confirmatory factor analysis and structural equation modeling analysis (SEM) was conducted by AMOS version 22.0. In SEM, a generalized least squares method was used because kurtosis of factors in the modified IMB model did not satisfy the normality assumption. The significance of indirect effects in the SEM was confirmed through bootstrapping. The fit indices for healthy behaviors for the metabolic syndrome model, the recommended levels of the Goodness of Fit Index (GFI), Adjusted Goodness of Fit Index (AGFI), Tucker Lewis Index (TLI), and Comparative Fit Index (CFI) were defined as ≥ .90, whereas the recommended level of RMSEA (Root Mean Square Error of Approximation) was defined as ≤ .08 [42]. The significance level was set at .05 with a two sided test.

## Results

### Characteristics of participants

The sociodemographic characteristics of study participants are presented in Table 1. Participants were 54.0 years old on average (*SD* = 8.1); about half of the sample were in their 50s (51.3 %). More women (54.3 %) than men (45.7 %) participated. More than 40 % of participants were high school graduates (41.6 %) and more than half were employed (53.9 %). The mean number of family members in the household was 3.1 (*SD* = 1.2) with only a few participants living alone (5.6 %). Time since being diagnosed with Mets was 1.83 years on average; the majority of participants had been diagnosed less than 1 year at the time of the study (64.4 %).

**Table 1:** Characteristics of study participants (*N* = 267)

Characteristic	Mean ± *SD*	*N*	%
Gender			
Male		122	45.7
Female		145	54.3
Age (years)	54.0 ± 8.1		
Education			
< High school		62	23.3
High school		111	41.6
> High school		94	35.1
Employment			
Employed		144	53.9
Unemployed		123	46.1
Time since diagnosis with metabolic syndrome (years)	1.8 ± 1.7		
<1		172	64.4
1–2		44	16.5
≥2		51	19.1

### Correlations among study variables

Correlations of information, psychological distress, motivation, behavioral skills, and practice of healthy behaviors for Mets are presented in Table 2. The practice of healthy behavior for Mets positively aligned with motivation (*r* = .44, *p* < .001) and behavioral skills for healthy behaviors (*r* = .51, *p* < .001), but negatively aligned with psychological distress (*r* = −.19, *p* = .004). The correlation coefficients of the variables for the modified IMB model of healthy behaviors for Mets ranged from .06 to .59, which was below the criterion of .80 for determining multicollinearity [43], and hence, no multicollinearity emerged among the variables.

**Table 2:** Correlations of variables in the modified Information-Motivation-Behavioral skill model for metabolic syndrome (*N* = 267)

Variable	Mean	SD	Range	Information	Psychological distress	Motivation	Behavioral skills
Information	11.6	2.3	0–14				
Psychological distress	2.0	0.7	1–5	−.19^*^			
Motivation	6.3	0.6	1–7	.30^***^	−.26^***^		
Behavioral skills	72.2	10.3	24–96	.13	−.23^***^	.59^***^	
Healthy behavior	91.0	18.5	35–140	.06	−.19^**^	.44^***^	.51^***^

^*^ *p* < .05; ^**^ *p* < .01; ^***^ *p* < .001

### Validation of the modified IMB model

**Fig. 2:**
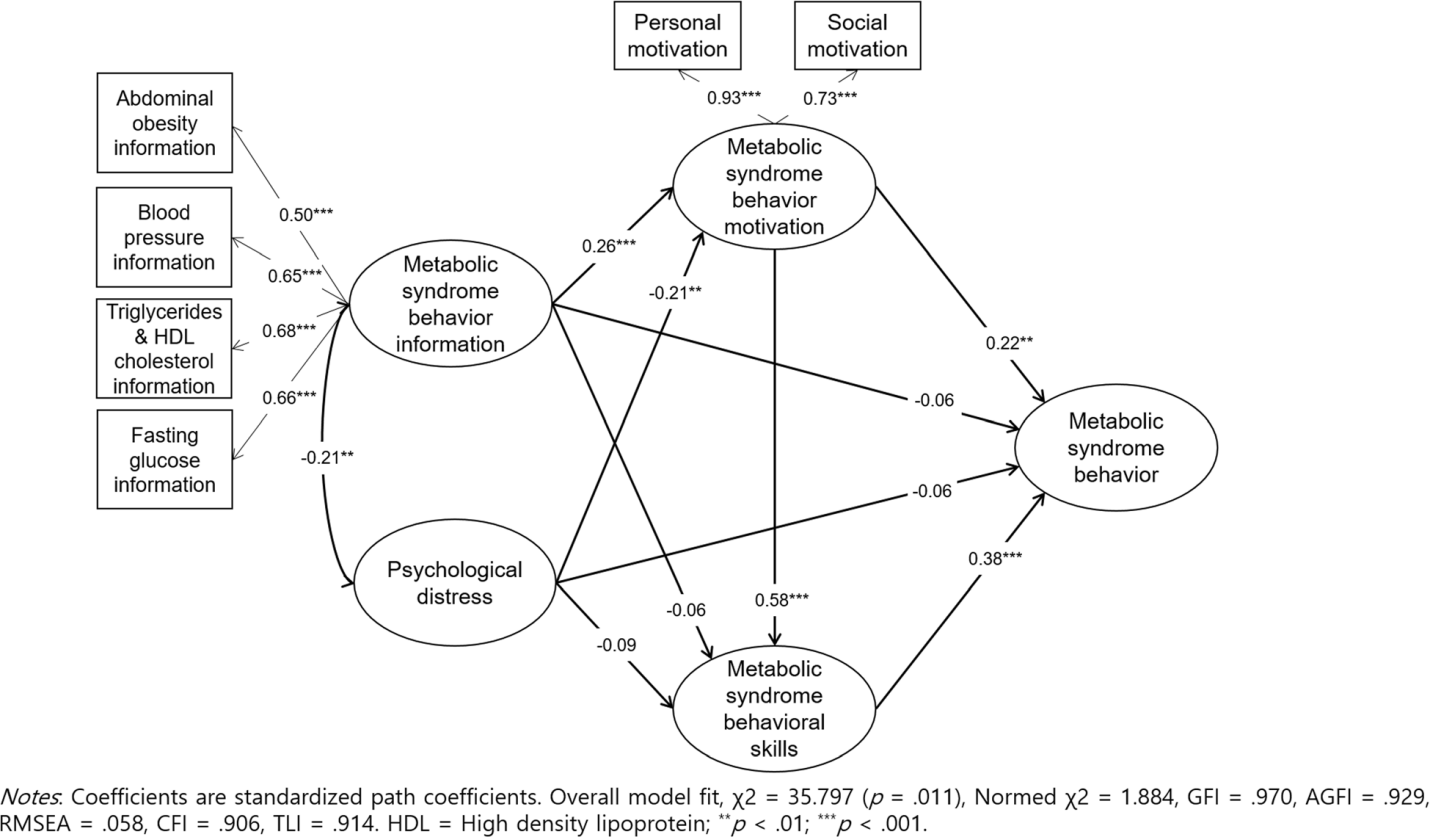
Estimation of the modified Information-Motivation-Behavioral skill model of metabolic syndrome healthy behavior

Prior to analysis of the modified IMB model using SEM, confirmatory factor analysis was conducted to examine the factor loadings structure to test the convergent validity of the observed variables of information and motivation. Factor loading distribution of observed variables on latent variables ranged from .50 to .93 (*p* < .001), which demonstrated good convergent validity [44] (Fig. 2).

The fitness indices for the modified IMB model in this study were *χ*^2^ = 35.797 (*p* = .011), Normed *χ*^2^ = 1.884, GFI = .970, AGFI = .929, RMSEA = .058, CFI = .906, and TLI = .914, showing the modified IMB model had good fit with the data. The explanatory power of the modified IMB model predicted 29.8 % of the practice of healthy behaviors for Mets. Information (*β* = .26, *p* < .001) and psychological distress (*β* = −.21, *p* = .001) significantly influenced motivation and influenced the practice of healthy behaviors for Mets through behavioral skills; motivation (*β* = .22, *p* = .005). Behavioral skills (*β* = .38, *p* < .001) also showed significant direct effects on the practice of healthy behaviors. Psychological distress and information did not have statistically significant direct effects on healthy behaviors for Mets.

## Discussion

This study examined the healthy behavior model of information deficits, motivational obstacles, and behavioral skills limitations in stress situations of individuals with Mets in Korea. The explanatory power of the modified model of healthy behaviors for Mets used in this study was found to be 29.8 %. Factors that directly influenced healthy behaviors were motivation and behavioral skills.

Although the effects of Mets information on healthy behaviors were not direct, information affected motivation, which positively influenced healthy behaviors in this study. In healthy behavior based interventions for patients with Mets, providing information helped strengthen the personal and social motivation perceived by participants [45, 46]. However, the effects of previous healthy behaviors interventions including providing information, depended on time, because education or periodical follow-ups could not influence lifestyle changes for Mets in such a short time [5, 17]. Behavioral changes may also be affected by time following diagnosis with Mets because health education is provided to those diagnosed with Mets; given that the majority of the participants were diagnosed with Mets within a year of this study, the effect of providing information on healthy behaviors may have been influenced by the amount of time since diagnosis with Mets and the particular characteristics of participants. More research is warranted to examine the differential impact of participant backgrounds on healthy behaviors for Mets.

Psychological distress did not impact healthy behaviors directly. However, distress did fully affect healthy behaviors indirectly through motivation and behavioral skills. Thus it may be hypothesized that, by managing psychological distress in patients with Mets, motivation and behavioral skills for healthy behaviors could be enhanced, which in turn, promotes healthy behaviors. In addition, during interventions for healthy behaviors for Mets, healthy behaviors that strengthen psychological distress management, such as following a healthy diet under stressful situations [14] and physical activities that include gradual muscle relaxation, which is effective for stress management [25] may be considered.

Direct effects of personal and social motivation on healthy behaviors were significant. These findings were similar to those from previous research [24, 47] that were reported favorable personal motivation positively influenced changing and maintaining healthy behaviors for Mets. Moreover, these findings were also similar to those in other studies (e.g., smoking cessation treatment) indicating that emotional support from family, friends, and peers promoted healthy behaviors [14, 25, 48, 49] or that informational support on detailed practices of healthy behaviors [50] strongly influences healthy behaviors for Mets.

Behavioral skills had the strongest direct effect on healthy behaviors in this study. This was similar to the findings in preceding studies that reported behavioral skills played an important role in practicing healthy behavior for Mets [15, 16]. Also, increased behavioral skills were related to improved healthy behaviors, such as balanced diet and increased physical activity [21, 51]. Also, behavioral strategies, such as repeating the sentence “I will walk at least 30 min” induced long term practices of healthy behaviors [14, 50]. In our IMB model, the practice of healthy behaviors for Mets should be promoted by considering information for healthy behavior [22] and Mets behavioral motivation including personal motivation [52] or social motivation [53].

This study was first to aim to identify a theoretical model that is appropriate to explain and predict healthy behaviors in patients in the community who suffer from Mets. Findings from this study have the potential to effect changes through healthy behaviors for Mets, especially in patients with Mets who do not take medications for diabetes or hypertension. In addition, healthy behaviors for Mets presented in this study added the psychological distress component to the IMB model. Through the analysis of its structural relationship, psychological distress was examined the mediating effects of motivation and behavioral skills factors in promoting healthy behaviors for Mets.

This study has limitations. The modified IMB model was confirmed through cross sectional data collection; therefore, one should be careful in interpreting causal relationships between the influencing factors of healthy behaviors for Mets. Also because it was the IMB model based study, variables such as health beliefs and behavior intention, which can also influence healthy behaviors, were not considered. Thus, caution should be taken in explaining healthy behaviors for Mets.

## Conclusions

It was confirmed that strengthening motivation and behavioral skills for healthy behaviors can directly enhance healthy behavior practice. Providing information on healthy behaviors for Mets and managing stress through the findings from this study can increase healthy behavior practice through motivation and behavioral skills for healthy behaviors. Effective intervention measures for healthy behaviors for Mets may be explored considering the relationships between these factors, and doing so will contribute to enhancing healthy behavior practice in patients with Mets, thereby preventing complications and improving quality of life of patients with Mets.

## Abbreviations

AGFI, Adjusted Goodness of Fit Index; CFI, Comparative Fit Index; GFI, Goodness of Fit Index; HDL, high-density lipoprotein; ICD, international classification of diseases; IMB, information-motivation-behavioral skills model; NFI, Normed Fit Index; RMSEA, Root-Mean-Square Error of Approximation; SEM, structural equation modeling

## References

[1] Grundy SM. Metabolic syndrome pandemic. Arterioscler Thromb Vasc Biol. 2008; doi:10.1161/ATVBAHA.107.151092. 10.1161/ATVBAHA.107.151092 18174459

[2] Kim EG, Oh SW (2012). Gender differences in the association of occupation with metabolic syndrome in Korean adults. Korean J Obes.

[3] World Health Organization, Departement of Noncommunicable Disease Surveillance (1999). The metabolic syndrome. Definition, diagnosis and classification of diabetes mellitus and its complications. Report of a WHO consultation.

[4] Alberti G, Zimmet P, International Diabetes Federation (2006). Worldwide definition for use in clinical practice. The IDF consensus worldwide definition of the metabolic syndrome.

[5] National Cholesterol Education Program (NCEP) Expert Panel on Detection, Evaluation, and Treatment of High Blood Cholesterol in Adults (Adult Treatment Panel III). Third Report of the National Cholesterol Education Program (NCEP) Expert Panel on Detection, Evaluation, and Treatment of High Blood Cholesterol in Adults (Adult Treatment Panel III) final report. Circulation. 2002;106:3143–421. 12485966

[6] Rask MC, Kahn CR (2012). Tissue-specific insulin signaling, metabolic syndrome and cardiovascular disease. Arterioscler Thromb Vasc Biol.

[7] Gami AS, Witt BJ, Howard DE, Erwin PJ, Gami LA, Somers VK (2007). Metabolic syndrome and risk of incident cardiovascular events and death: a systematic review and meta-analysis of longitudinal studies. J Am Coll Cardiol.

[8] Sullivan VK (2006). Prevention and treatment of the metabolic syndrome with lifestyle intervention: where do we start?. J Am Diet Assoc.

[9] Grundy SM, Cleeman JI, Merz CN, Brewer HB, Clark LT, Hunninghake DB (2004). Implications of recent clinical trials for the National Cholesterol Education Program Adult Treatment Panel III Guidelines. J Am Coll Cardiol.

[10] Glanz K, Kirscht JP, Rosenstock IM (1981). Linking research and practice in patient education for hypertension: patient responses to four educational interventions. Med Care.

[11] Sturt J, Taylor H, Docherty A, Dale J, Louise T (2006). A psychological approach to providing self-management education for people with type 2 diabetes: the diabetes manual. BMC Fam Pract.

[12] Murer M, Schmied C, Battegay E, Keller DI. Physical activity behaviour in patients with metabolic syndrome. Swiss Med Wkly. 2012; doi:10.4414/smw.2012.13691. 10.4414/smw.2012.13691 23136010

[13] Kudo Y, Okada M, Tsunoda M, Satoh T, Aizawa Y (2011). A lifestyle to prevent or combat the metabolic syndrome among Japanese workers: analyses using the health belief model and the multidimensional health locus of control. Ind Health.

[14] Foreyt JP (2005). Need for lifestyle intervention: how to begin. Am J Cardiol.

[15] Mohebi S, Azadbakht L, Feizi A, Sharifirad G, Hozori M. Predicting of perceived self-efficacy in the amount of macronutrients intake in women with metabolic syndrome–2012. J Educ Health Promot. 2014; doi:10.4103/2277-9531.127608. 10.4103/2277-9531.127608 PMC3977411 24741661

[16] Strecher VJ, DeVellis BM, Becker MH, Rosenstock IM (1986). The role of self-efficacy in achieving health behavior change. Health Educ Q.

[17] Yamaoka K, Tango T. Effects of lifestyle modification on metabolic syndrome: a systematic review and meta-analysis. BMC Med. 2012; doi:10.1186/1741-7015-10-138. 10.1186/1741-7015-10-138 PMC3523078 23151238

[18] Ajzen I (1991). The theory of planned behavior. Organ Behav Hum Decis Process.

[19] Pender NJ, Walker SN, Sechrist KR, Stromborg MF (1988). Development and testing of the health promotion model. Cardiovasc Nurs.

[20] Fisher JD, Fisher WA (1992). Changing AIDS-risk behavior. Psychol Bull.

[21] Dalle GR, Calugi S, Centis E, Marzocchi R, El GM, Marchesini G (2010). Lifestyle modification in the management of the metabolic syndrome: achievements and challenges. Diabetes Metab Syndr Obes.

[22] Chen AM, Yehle KS, Albert NM, Ferraro KF, Mason HL, Murawski MM (2014). Relationships between health literacy and heart failure knowledge, self-efficacy, and self-care adherence. Res Social Adm Pharm.

[23] Fisher WA, Fisher JD, Harman J, Suls J, Wallston KA (2003). The information-motivation-behavioral skills model: a general social psychological approach to understanding and promoting health behavior. Social psychological foundations of health and illness.

[24] Lin CH, Chiang SL, Tzeng WC, Chiang LC (2014). Systematic review of impact of lifestyle-modification programs on metabolic risks and patient-reported outcomes in adults with metabolic syndrome. Worldviews Evid Based Nurs.

[25] Foreyt JP, Poston WS (2000). Successful management of the obese patient. Am Fam Physician.

[26] Isomaa B (2003). A major health hazard: the metabolic syndrome. Life Sci.

[27] Newcomer JW, Selke G, Melson AK, Gross J, Vogler GP, Dagogo JS (1998). Dose-dependent cortisol-induced increases in plasma leptin concentration in healthy human. Arch Gen Psychiatry.

[28] Björntorp P (2001). Heart and soul: stress and the metabolic syndrome. Scand Cardiovasc J.

[29] Kim CJ (2010). Mental health and self-care activities according to perceived stress level in type 2 diabetic patients with metabolic syndrome. J Korean Acad Adult Nurs.

[30] Ellis DA, Janisse H, Naar-King S, Kolmodin K, Jen KL, Cunningham P (2010). The effects of multisystemic therapy on family support for weight loss among obese African-American adolescents: findings from a randomized controlled trial. J Dev Behav Pediatr.

[31] McKenzie SH, Harris MF. Understanding the relationship between stress, distress and healthy lifestyle behaviour: a qualitative study of patients and general practitioners. BMC Fam Pract. 2013; doi:10.1186/1471-2296-14-166. 10.1186/1471-2296-14-166 PMC3817353 24175998

[32] Seeman TE (2000). Health promoting effects of friends and family on health outcomes in older adults. Am J Health Promot.

[33] Kelly RB, Zyzanski SJ, Alemagno SA (1991). Prediction of motivation and behavior change following health promotion: role of health beliefs, social support, and self-efficacy. Soc Sci Med.

[34] Oh EG, Bang SY, Hyun SS, Chu SH, Jeon JY, Kang MS (2007). Knowledge, perception and health behavior about metabolic syndrome for an at risk group in a rural community area. J Korean Acad Nurs.

[35] Frank SH, Zyzanski SJ (1988). Stress in the clinical setting: the Brief Encounter Psychosocial Instrument. J Fam Pract.

[36] Yim JH, Bae JM, Choi SS, Kim SW, Hwang SH, Huh BY (1996). The validity of modified Korean-translated Brief Encounter Psychosocial Instrument as instrument of stress measurement in outpatient clinic. J Korean Acad Fam Med.

[37] Ajzen I, Madden TJ (1986). Prediction of goal-directed behavior: attitudes, intentions and perceived behavioral control. J Exp Soc Psychol.

[38] Francis JJ, Eccles MP, Johnston M, Walker A, Grimshaw J, Foy R, et al. Constructing questionnaires based on the theory of planned behavior: a manual for health services researchers. Centre for Health Services Research, University of Newcastle upon Tyne. 2004. http://openaccess.city.ac.uk/id/eprint/1735. Accessed 16 Jun 2016.

[39] Kang SW (2010). The validity and reliability of a lifestyle evaluation tool for patients with metabolic syndrome. J Korean Acad Fundam Nurs.

[40] Becker H, Stuifbergen H, Oh HS, Hall S (1993). Self-rated abilities for health practices: a health self-efficacy measure. Health Values.

[41] Choi JM, Moon IO (2005). The effects of college students’ self-efficacy on their health promotion behavior. Korean Pub Health Res.

[42] Fornell C, Larcker DF (1981). Evaluating structural equation models with unobservable variables and measurement error. J Marketing Res.

[43] Bollen KA (1989). Structural equations and latent variables.

[44] Bagozzi RP, Yi Y (1989). On the evaluation of structural equation models. J Acad Market Sci.

[45] Bull S, Eakin E, Reeves M, Kimberly R (2006). Multi-level support for physical activity and healthy eating. J Adv Nurs.

[46] Frisman GH, Berterö C (2008). Having knowledge of metabolic syndrome: does the meaning and consequences of the risk factors influence the life situation of Swedish adults?. Nurs Health Sci.

[47] Parker WA, Steyn NP, Levitt NS, Lombard CJ (2011). They think they know but do they? Misalignment of perceptions of lifestyle modification knowledge among health professionals. Public Health Nutr.

[48] Helgason AR, Tomson T, Lund KE, Galanti R, Ahnve S, Gilljam H (2004). Factors related to abstinence in a telephone helpline for smoking cessation. Eur J Public Health.

[49] Bezares VR, Bacardí GM, Márquez RS, Molinero GO, Estrada GM, Jiménez CA (2013). Efficacy of social support on metabolic syndrome among low income rural women in Chiapas. México Nutr Hosp.

[50] Mechanic D, Cleary PD (1980). Factors associated with the maintenance of positive health behavior. Prev Med.

[51] Choi SH, Choi KS (2015). The effects of the DASH diet education program with omega-3 fatty acid supplementation on metabolic syndrome parameters in elderly women with abdominal obesity. Nutr Res Pract.

[52] Backman DR, Haddad EH, Lee JW, Johnston PK, Hodgkin GE (2002). Psychosocial predictors of healthful dietary behavior in adolescents. J Nutr Educ.

[53] Trouillet R, Gana K, Lourel M, Fort I (2009). Predictive value of age for coping: the role of self-efficacy, social support satisfaction and perceived stress. Aging Ment Health.

